# Supplementary material for: Case of Early-Onset Parkinson’s Disease in a Heterozygous Mutation Carrier of the ATP7B Gene
Source: J Pers Med. 2019 Aug 17;9(3):41. doi: 10.3390/jpm9030041 (PMC6789574; doi:10.3390/jpm9030041)
Supplement: Supplementary file 1 [file jpm-09-00041-s001.pdf]

Journal of Personalized Medicine

MDPI

Table S1. MM-GBSA free energy per residue pair decomposition (ΔG, kcal/mol).

Residue	WT	C1079F	C1079G
Val1039	-1.3 ± 0.2	-0.2 ± 0.1	0.0 ± 0.0
Val1042	-0.9 ± 0.3	-0.8 ± 0.2	0.0 ± 0.0
Arg1054	-0.0 ± 0.0	-2.8 ± 0.6	-0.9 ± 0.6
Leu1057	-0.1 ± 0.0	-2.7 ± 0.5	-1.0 ± 0.2
Ala1058	-0.0 ± 0.0	-1.1 ± 0.2	-0.7 ± 0.2
Val1060	-1.0 ± 0.3	-0.0 ± 0.0	0.0 ± 0.0
Val1075	-2.3 ± 0.4	-2.7 ± 0.6	-1.6 ± 0.4
Thr1076	-2.1 ± 0.3	-2.3 ± 0.4	-1.7 ± 0.3
Lys1077	-1.0 ± 0.2	-1.2 ± 0.2	-0.9 ± 0.2
Tyr1078	-12.0 ± 0.4	-13.6 ± 0.7	-10.6 ± 0.4
Lys1080	-4.9 ± 0.4	-5.3 ± 0.4	-5.5 ± 0.4
Glu1081	-0.9 ± 0.1	-0.9 ± 0.1	-0.9 ± 0.2
Glu1082	-1.9 ± 0.3	-2.2 ± 0.3	-1.8 ± 0.4
Leu1083	-2.0 ± 0.4	-2.5 ± 0.6	-2.5 ± 0.4

The residues in the table revealed the free energy of contact with the residue in position 1079 lower than 0.6 kcal/mol (~RT, T=300 K) at least in one of the simulations.

Table S2. MM-GBSA free energy per residue decomposition (ΔG, kcal/mol).

Residue	WT	C1079F	C1079G
Res1079	13.3 ± 2.0	14.0 ± 3.2	1.1 ± 1.5
Val1039	-13.0 ± 2.9	-11.7 ± 2.8	-12.2 ± 3.0
Val1042	-13.0 ± 2.9	-11.1 ± 2.5	-10.6 ± 2.9
Arg1054	-164.9 ± 3.2	-163.5 ± 3.3	-164.5 ± 3.4
Leu1057	-14.6 ± 3.4	-15.2 ± 2.7	-15.2 ± 2.7
Ala1058	3.1 ± 1.9	4.3 ± 2.2	3.2 ± 2.0
Val1060	-9.2 ± 2.5	-11.1 ± 2.5	-11.3 ± 2.5
Val1075	-11.5 ± 2.3	-9.3 ± 2.7	-10.3 ± 2.9
Thr1076	-15.4 ± 2.7	-15.2 ± 2.5	-14.0 ± 2.4
Lys1077	-1.0 ± 0.2	-1.2 ± 0.2	-0.9 ± 0.2
Tyr1078	-15.5 ± 3.5	-15.3 ± 3.1	-14.1 ± 3.1
Lys1080	-15.7 ± 3.6	-18.3 ± 3.8	-16.8 ± 3.6
Glu1081	-58.3 ± 2.8	-58.5 ± 2.6	-57.6 ± 3.1
Glu1082	-49.6 ± 2.6	-49.7 ± 3.0	-49.9 ± 2.6
Leu1083	-15.8 ± 2.5	-12.4 ± 2.6	-14.1 ± 2.5

The residues in the table revealed the free energy of contact with the residue in position 1079 lower than 0.6 kcal/mol (~RT, T=300 K) at least in one of the simulations.
